# The effects of antibiotic cycling and mixing on acquisition of antibiotic resistant bacteria in the ICU: A post-hoc individual patient analysis of a prospective cluster-randomized crossover study

**DOI:** 10.1371/journal.pone.0265720

**Published:** 2022-05-03

**Authors:** Pleun J. van Duijn, Walter Verbrugghe, Philippe G. Jorens, Fabian Spöhr, Dirk Schedler, Maria Deja, Andreas Rothbart, Djillali Annane, Christine Lawrence, Matjaz Jereb, Katja Seme, Franc Šifrer, Viktorija Tomič, Francisco Estevez, Jandira Carneiro, Stephan Harbarth, Marc J. M. Bonten

**Affiliations:** 1 Department of Epidemiology, Julius Center for Health Sciences and Primary Care, Utrecht, Netherlands; 2 Department of Critical Care Medicine, Antwerp University Hospital, University of Antwerp, Edegem, Belgium; 3 Department of Anaesthesiology and Intensive Care, Sana Kliniken Stuttgart, Stuttgart, Germany; 4 Klinik für Anästhesiologie und Intensivmedizin, Universitätsklinikum Schleswig-Holstein, Lübeck, Germany; 5 Department of Anesthesiology and Operative Intensive Care Medicine, Charité Universitätsmedizin, Berlin, Germany; 6 Department of Intensive Care, Hôpital Raymond Poincaré (APHP), Garches, France; 7 Laboratory of Infection & Inflammation, School of Medicine Simone Veil, University Versailles Saint Quentin & University Paris Saclay, INSERM, Montigny le Bretonneux, France; 8 FHU SEPSIS (Saclay and Paris Seine Nord Endeavour to PerSonalize Interventions for Sepsis) and RHU RECORDS (Rapid rEcognition of CORticosteroiD resistant or sensitive Sepsis), Garches, France; 9 Microbiology Unit, Raymond-Poincaré Hospital, Garches, France; 10 Department of Infectious Diseases, University Medical Centre, Ljubljana, Slovenia; 11 Medical Faculty, University of Ljubljana, Ljubljana, Slovenia; 12 Faculty of Medicine, Institute of Microbiology and Immunology, University of Ljubljana, Ljubljana, Slovenia; 13 Department of Intensive Care Medicine, University Hospital Golnik, Golnik, Slovenia; 14 Department of Medical Microbiology, University Hospital Golnik, Golnik, Slovenia; 15 Intensive Care Unit and Emergency Department, Centro Hospitalar Trás-os-Montes e Alto Douro, Vila Real, Portugal; 16 Infection Control Program, Geneva University Hospitals and Faculty of Medicine, Geneva, Switzerland; 17 Department of Medical Microbiology, University Medical Center Utrecht, Utrecht, Netherlands; Rabin Medical Center, Beilinson Hospital, ISRAEL

## Abstract

**Background:**

Repeated rotation of empiric antibiotic treatment strategies is hypothesized to reduce antibiotic resistance. Clinical rotation studies failed to change unit-wide prevalence of antibiotic resistant bacteria (ARB) carriage, including an international cluster-randomized crossover study. Unit-wide effects may differ from individual effects due to “ecological fallacy”. This post-hoc analysis of a cluster-randomized crossover study assesses differences between cycling and mixing rotation strategies in acquisition of carriage with Gram-negative ARB in individual patients.

**Methods:**

This was a controlled cluster-randomized crossover study in 7 ICUs in 5 European countries. Clinical cultures taken as routine care were used for endpoint assessment. Patients with a first negative culture and at least one culture collected in total were included. Community acquisitions (2 days of admission or less) were excluded. Primary outcome was ICU-acquisition of Enterobacterales species with reduced susceptibility to: third- or fourth generation cephalosporins or piperacillin-tazobactam, and Acinetobacter species and *Pseudomonas aeruginosa* with reduced susceptibility for piperacillin-tazobactam or carbapenems. Cycling (altering first-line empiric therapy for Gram-negative bacteria, every other 6-weeks), to mixing (changing antibiotic type every empiric antibiotic course). Rotated antibiotics were third- or fourth generation cephalosporins, piperacillin-tazobactam and carbapenems.

**Results:**

For this analysis 1,613 admissions were eligible (855 and 758 during cycling and mixing, respectively), with 16,437 microbiological cultures obtained. Incidences of acquisition with ARB during ICU-stay were 7.3% (n = 62) and 5.1% (n = 39) during cycling and mixing, respectively (p-value 0.13), after a mean of 17.7 (median 15) and 20.8 (median 13) days. Adjusted odds ratio for acquisition of ARB carriage during mixing was 0.62 (95% CI 0.38 to 1.00). Acquired carriage with ARB were Enterobacterales species (n = 61), *Pseudomonas aeruginosa* (n = 38) and Acinetobacter species (n = 20), with no statistically significant differences between interventions.

**Conclusions:**

There was no statistically significant difference in individual patients’ risk of acquiring carriage with Gram-negative ARB during cycling and mixing. These findings substantiate the absence of difference between cycling and mixing on the epidemiology of Gram-negative ARB in ICU.

**Trial registration:**

This trial is registered with ClinicalTrials.gov, registered 10 January 2011, NCT01293071.

## Introduction

Treatment of critically ill patients admitted in Intensive Care Units (ICU) is frequently complicated by infections caused by antibiotic resistant Gram-negative bacteria. Patients in ICU often receive broad spectrum antibiotics which increases antibiotic resistance selective pressure and the chance of acquiring colonization with Gram-negative antibiotic resistant bacteria (ARB). To reduce this selective pressure, unit-wide antibiotic stewardship programs (ASP) have been advocated, sometimes advocating but also discouraging antibiotic rotation strategies [1–3]. These strategies aim to modulate the diversity of antibiotic exposure in a ward, rather than reducing overall antibiotic use. The increased heterogeneity of antibiotic exposure, hypothetically, reduces antibiotic resistance selection pressure and occurrence of antibiotic resistant bacteria [4–12].

Previous observational and quasi-experimental studies, however, yielded non-conclusive results for different pathogens, rotation schedules and outcomes [13–26]. In a multi-center cluster crossover study, two antibiotic rotation interventions, cycling and mixing, we found similar effects on the unit-wide ecology of Gram-negative ARB [27]. In that study two different antibiotic rotation strategies for empiric treatment of patients with presumed Gram-negative infections were compared: During cycling the preferred antibiotic treatment changed every six weeks and during mixing it changed after every single patient. Effectiveness of the intervention was determined by measuring the prevalence of carriage with antibiotic resistant Gram-negative bacteria at the unit level, through monthly point-prevalence surveys. However, group-ecological and individual risks can differ, even within the same experimental study, due to what is called the “ecological fallacy” [28]. We, therefore, performed a post-hoc analysis on study data of this previously performed study to investigate whether two antibiotic rotation schemes, cycling and mixing, yielded differences in the individual risk of acquiring Gram-negative ARB during ICU admission.

## Methods

### Study design

This was a post-hoc nested cohort analysis of a cluster-randomized cross-over study [29]. Four months of standard care treatment preceded ICU randomization to two 9-month intervention periods. A one-month wash-out period separated the intervention periods. ICUs were cluster-allocated by randomization to perform either the cycling strategy followed by mixing or vice versa. Computer randomization of the allocation to interventions (in a 1:1 ratio) and randomization of the order of consecutively rotated antibiotics (in a random sequence per ICU) was performed by a person not involved in designing or performing the study. First-line empirical therapy for patients with assumed infections that required treatment of Gram-negative bacteria was rotated in 6-week periods between 3rd or 4th-generation cephalosporins, piperacillin-tazobactam and carbapenems (cycling). During mixing, the preferred empiric therapy was rotated for every new patient needing treatment. Patients could be treated with different antibiotic courses during admission. Readmissions were included. To safeguard optimal patient care, protocol allowed for physicians to change patient therapy on an individual basis at any time (e.g., de-escalation, combination therapy, allergic reactions or patient safety). There was no blinding of intervention allocation for physicians during admission, for those responsible for data collection or the patient. For the current analysis we had access to individual microbiological culture data from seven of eight participating European ICUs from Belgium, France, Germany, Portugal, and Slovenia. These were ICUs with mixed, medical or surgical patient populations.

Patients were included if a first culture was negative for Gram-negative ARB in cultures from the respiratory- or gastrointestinal tract (e.g. feces, rectum, perineum or gastric contents).

Enrolment of patients was preceded by approvement of the study and a waiver for individual informed consent, by all local Interne Review Boards of each participating center. This trial is registered with ClinicalTrials.gov, number NCT01293071. There were no changes to the study design after trial commencement.

The primary endpoint was the first clinical culture with Gram-negative ARB, defined as non-susceptibility to 3^rd^- or 4^th^ generation cephalosporins and piperacillin-tazobactam in Enterobacterales species, and piperacillin-tazobactam or- meropenem resistance in *Pseudomonas aeruginosa* and Acinetobacter species. For the current analysis patients were eligible if microbiological cultures had been obtained from either the respiratory tract or gastrointestinal tract. Admissions with detected Gram-negative ARB carriage in the first two days of admission were excluded, as were admissions with positive endpoints on the same day as a first negative culture. Acquired carriage with Gram-negative ARB after day 2 of admission was assumed to be permanent for the duration of the study, i.e., readmissions of these patients were excluded from the analysis.

### Data collection

All clinical cultures taken during the study periods were included for endpoint analysis. If the primary endpoint was reached in an individual patient, subsequent culture results were excluded. Microbiological procedures were performed according to local laboratory practices, including local Minimum Inhibitory Concentration cutoff values. Participating microbiological laboratories did not change protocols for antibiotic susceptibility testing during the study. Likewise, infection prevention- and control measures did not change during the study period.

Systemic antibiotic use was collected at the individual or aggregate level. Consumed quantities of antibiotics were converted to WHO Defined Daily Dose (DDD). Use of different antibiotic groups were represented as subdivided 6-week periods for cycling, and divided over the 9-month intervention period for mixing.

### Ethics approval and consent to participate

The study protocol was approved by each local Institutional Review Board (IRB) and all centers obtained a waiver for individual patient written informed consent.

### Statistical analysis

Demographic- and infection prevention variables comparison between interventions were performed using bivariate tests. Dichotomous endpoints were tested using Pearsons’ chi-square test and for continuous outcomes using Students’ t-test. The primary outcome analysis was performed using chi-square test for binary endpoints. Odds ratios for acquisition between the two intervention periods (cycling and mixing) were calculated using mixed effects logistic regression modelling, with adjustment for clustering within each hospital and for confounders age, gender, length of stay, previous admission, origin of transfer to ICU and survival at ICU-discharge). Independent variables for this model were chosen based on potential correlation with the intervention and endpoint and being reasonably objectifiable.

Additionally, detection bias between intervention periods from clinical cultures was assessed by modeling the probability of having a culture taken during admission using a mixed effects model correcting for clustering within individual ICU. This analysis was performed on all admissions during the intervention period, including patients without clinical cultures obtained. To assess competing events bias, mean and median length of stay was calculated and linear regression modelling of the effect of intervention type on length of stay.

Carryover effects between first and second intervention period were assessed using the mixed effects model with an additional interaction term between intervention type and the sequence of performing mixing-then-cycling or vice-versa.

Post-hoc power calculations were performed using the *pwr* package, an effect size of 0.1 based on an arbitrary ‘small’ effect, significance level of 5% and assumed a two-sided alternative outcome. The calculated power to find a relevant difference was 88%. Analyses were performed using R *software* [30].

## Results

**Fig 1:**
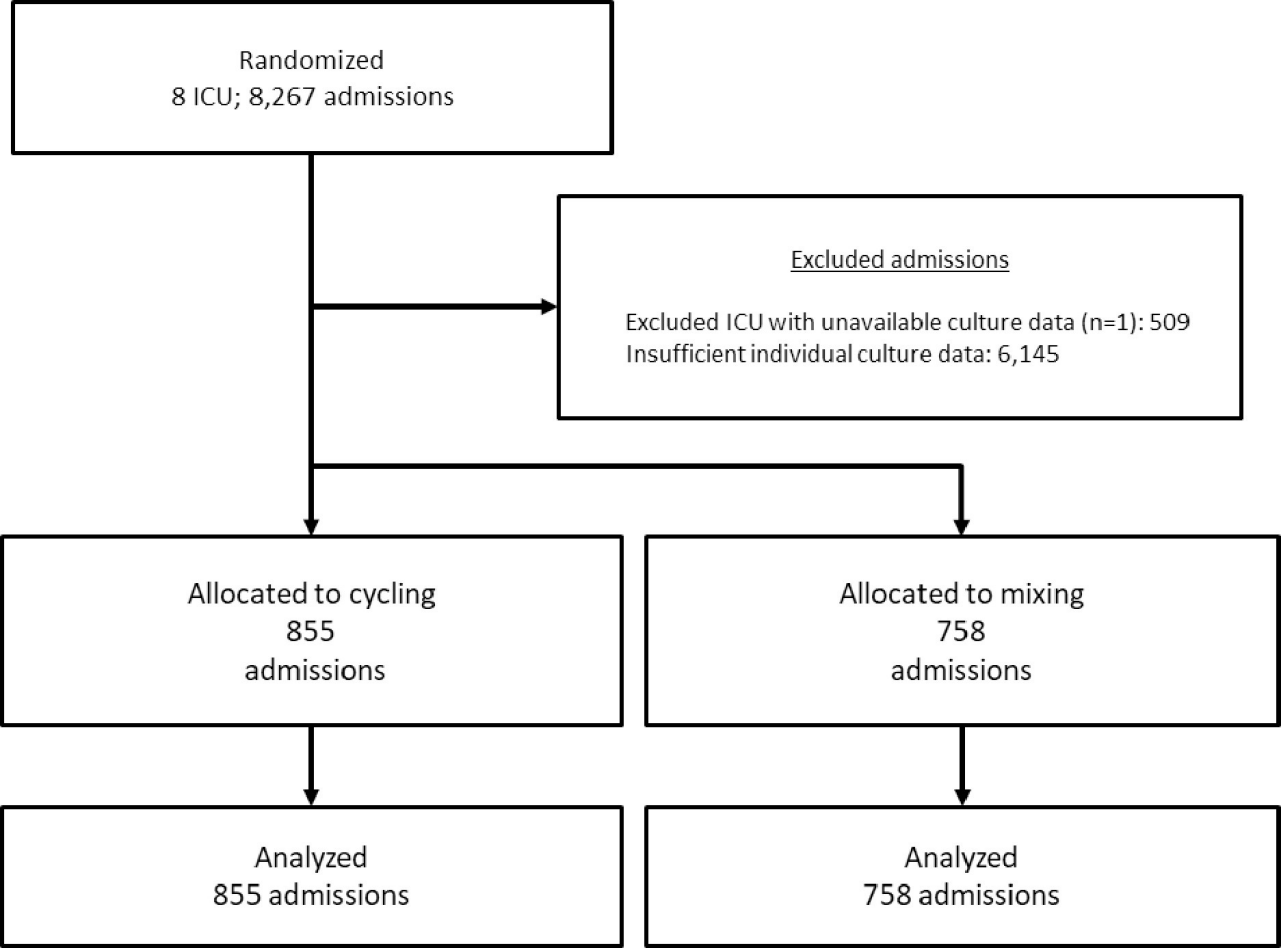
Flow chart.

During the cycling and mixing intervention periods, there were 8,267 admissions overall in 7 ICUs in 5 countries (Fig 1). For this nested cohort study, 1,613 (19.5%) admissions were eligible. Data was collected from June 27, 2011, to February 16, 2014. Baseline demographics and ICU characteristics of interventions were comparable (Tables 1 and 2).

**Table 1:** Demographic data.

Demographic variables	Total
Total Admissions	1,613
Male % (N)	60.8 (980)
Age mean (median)	61.2 (63.8)
Length of stay mean (median)	11.5 (6)
Short-stay patients (< = 2days)^*a*^ % (N)	15.4 (248)
Mortality % (N)	12.3 (198)
APACHEII mean (median, N) [31]	19.9 (19; 408)
APACHEII (N hospitals)	3
SAPSII mean (median, N)	36.6 (33; 544)
SAPSII (N hospitals)	5
SAPSIII mean (median, N)	55.6 (54; 278)
SAPSIII (N hospitals)	2
TIS28 mean (median, N)	24.3 (24; 160)
TIS28 (N hospitals)	2

^*a*^ Patients with a LOS of 2 days or less

**Table 2:** Overall ICU characteristics, all admissions during study periods.

ICU characteristic, mean	Cycling	Mixing	p-value
Bed occupancy % (beds taken/available)	77.3 (734/949)	80.4 (867/1,079)	*0*.*59*^a^
Mechanically ventilated patients % (N)	48.8 (358)	42.2 (366)	*0*.*24*^a^
CVVH % (N)^b^	4.6 (34)	4.8 (42)	*0*.*94*^a^
ECMO % (N)^c^	3.0 (22)	4.8 (42)	*0*.*09*^a^
Thoracic drains % (N)	16.8 (123)	16.5 (143)	*0*.*96*^a^
Abdominal drains % (N)	12.3 (90)	8.2 (71)	*0*.*02*^a^
Intra-cranial pressure monitors % (N)	1.9 (14)	1.9 (16)	*1*.*00*^a^
Contact isolation % (N)	24.9 (183)	24.8 (215)	*1*.*00*^a^
Droplet isolation % (N)	1.6 (12)	2.2 (19)	*0*.*54*^a^
Airborne isolation % (N)	1.0 (7)	1.7 (15)	*0*.*27*^a^
Staffing ratio (registered nurses/1 patient)	0.66	0.64	*0*.*67*^*d*^
Staffing ratio (student nurse/1 patient)	0.11	0.10	*0*.*74*^*d*^

^*a*^ Pearson Chi square

^b^ Continuous Veno-Venous Hemofiltration Dialysis

^c^ Extra-Corporeal Membrane Oxygenation

^d^ Student t-test, two-sided

In these patients 16,437 microbiological cultures were collected (mean of 10.27 cultures per admission); upper- or lower respiratory materials (%), blood- or intravascular cultures (34.8%), enteric cultures (e.g. gastric fluid, bile, feces, rectum swabs, 29.9%) and urine (12.2%) (Appendix Table 1 in S1 Appendix). In total 8.3% of the cultures was taken as part of surveillance for carriage, not for diagnosis and treatment of infection (missings 12.7%). In cultures that grew *Enterobacterales*, most were *Escherichia coli* (22.5%), Pseudomonas species (21.2%), Klebsiella species (18.8%) and Enterobacter species (11.2%, Appendix Table 2 in S1 Appendix). The odds ratio (OR) of having a culture taken was lower during the mixing intervention (OR 0.83, 95% confidence interval (CI) 0.76 to 0.91, p-value <0.01) (Appendix Table 3 in S1 Appendix), which did not change after correction for confounders (age, gender, length of stay, previous admission and origin of transfer/referral): Adjusted odds ratio (aOR) 0.84 (95% CI 0.76 to 0.93).

In all, there were 855 and 758 admissions eligible for analysis during cycling and mixing, respectively. The primary endpoint was reached in 62 (7.3%) and in 39 (5.1%) patients during cycling and mixing, respectively, (p-value: 0.13), after a mean of 17.7 (median 15) and 20.8 (median 13) days, in cycling and mixing respectively (p-value: 0.43). Distributions of endpoint defined micro-organisms were: Enterobacterales species (n = 61), *Pseudomonas aeruginosa* (n = 38) and Acinetobacter species (n = 20), without statistically significant differences between interventions (Table 3).

**Table 3:** Acquisition of ARB (primary endpoint).

Acquisition variables		Cycling	Mixing	p-value^a^
Included clinical cultures N^b^		9,236	7,201	
Included admissions N (%)		855 (21.9)	758 (17.4)	*<0*.*01*
Number of cultures per patient	mean (SE)	10.6 (0.50)	9.2 (0.43)	*0*.*03*
	median (range)	5 (1–129)	5 (1–117)	
Admissions with ≥1 ARB endpoints N (%)^c^		62 (7.3)	39 (5.1)	*0*.*13*
Enterobacterales species resistance endpoint N (%)		38 (4.4)	23 (3.0)	*0*.*18*
*Pseudomonas aeruginosa* resistance endpoint N (%)		25 (2.9)	13 (1.7)	*0*.*15*
Acinetobacter species resistance endpoint N (%)		10 (1.2)	10 (1.3)	*0*.*96*
Days from admission till first negative culture	mean (SE)	3.1 (0.09)	2.7 (0.05)	*<0*.*01*
	median (range)	2 (2–28)	2 (2–14)	
Days from first negative culture till endpoint	mean (SE)	12.1 (1.4)	15.1 (3.1)	*0*.*39*
	median (range)	9 (1–49)	8 (1–108)	

^*a*^ Chi-square test for binary variables and T-test for continuous variables.

^b^ Cultures of patients with >1 culture, of which the first was negative, excluding endpoints < = 2 days admission

^c^ Aggregated endpoint of Enterobacterales species, Pseudomonas aeruginosa and/or Acinetobacter species endpoints

SE = Standard error

Antibiotic use per interventions were similar, as expected by the protocol of equal use of each antibiotic (group) type over time (Appendix Table 4 in S1 Appendix).

In mixed effect logistic regression modelling the unadjusted odds ratio for acquiring Gram-negative ARB was 0.72 (CI 95% 0.47 to 1.10) during mixing compared to cycling (Table 4). After adjustment for age, gender, length of stay, previous admission and origin of referral (community or hospital), adjusted odds ratio was 0.62, 95% CI 0.38 to 1.00). Adding an interaction term for intervention strategy with the sequence of strategies to test for a carryover effect did not change results (Appendix Table 5 in S1 Appendix).

**Table 4:** Mixed effects logistic regression odds ratios.

Analysis type	Model type	Mixing:cycling odds ratio	Confidence interval (2.5%-97.5%)	p-value
**Primary *analysis*** ^***a***^	**Unadjusted *model*** ^***b***^	0.72	0.47 till 1.11	0.13
	**Adjusted *model*** ^***c***^	0.62	0.38 till 1.00	0.05

^*a*^ Patients with >1 clinical culture taken with the first culture negative

^*b*^ Random effect: Hospital

^*c*^Adjusted variables: Age, Gender, LOS, Previous admission, Community of hospital referral, random effect: Hospital

The culture probability OR was used for a sensitivity analysis. Here we assume the mixing population was indeed cultured less with missed endpoints. We assumed the size of this hypothetical mixing population was equal to the ‘real’ cultured cycling population. With an OR of 0.83, the mixing hypothetical population is roughly 120% of the real mixing population. To meet statistical significance in a comparison of the hypothetical mixing population and the real cycling population, the incidence in this missed group would need to be 17%. This is more than two times the prevalence found in the real mixing study population.

## Discussion

In this patient-level analysis of a randomized cluster design multi-center study, we found no difference in effect of two antibiotic rotation strategies on the individual risk of acquiring colonization with Gram-negative ARB. These findings are in line with the results of the previously reported ecological analysis of this trial [29].

Cycling and mixing are strategies that create opposite extremes of antibiotic diversity: Cycling maximizes homogeneity during a 6-week period, and mixing maximizes heterogeneity.

The cluster randomized, crossover design allows comparison of unit-wide interventions in ICUs. As compared to cohort studies it reduces the likelihood of differences in clinical practice between ICUs, and other differences such as case-mix that may affect endpoint risk estimates. Compared to individual randomization, cluster randomization prevents non-adherence to protocol in individual patients, but provides an estimate of intervention effect at the ward level.

With respect to patient characteristics there were statistically significant differences in mortality and illness severity scores. For mortality this was assumed to be a chance finding, as both intervention arms did not aim to influence mortality, and both arms received the same amounts and types of antibiotics. This difference is also reflected in the mortality scores APACHE II, SAPS II, TIS-28, which do not represent relevant differences: 2, 4.1 and 4.8 respectively points on these scores with maximum scores being from 55, 78 to 163.

It was not possible to compare the interventions to a non-intervention period due to high baseline variability of antibiotic use between ICUs. More specific, there is no parameter to measure antibiotic diversity, therefore it is not possible to quantitatively compare baseline periods, whether these are alike -or different- to their corresponding mixing or cycling periods.

This post-hoc analysis was motivated by previous findings of different results from ecological- and individual-based analyses [28]. This study analyzed individual risks ratio of acquisition of ARB, in contrast to the previous ecological study that evaluated aggregated monthly point-prevalence surveys. Though ecological analyses offer useful information on group-wise interventions, such analyses can omit patient-level causal relations between exposure and outcome. This can lead to a form of bias called the ecological fallacy. Consequently, it has been advised to use both ecological as well as individual-level analyses for studies in antibiotic resistance when possible [28].

Ecological fallacy occurs when group-averaged risks lead to incorrect associations between exposure and outcome. For instance, when the exposed patients are not the same patients that acquire the endpoint. Or, when group analyses overlook that the outcome might have occurred before the exposure, distorting causal inference. Or when individual baseline and longitudinal risks differ over time, creating different subgroup risk profiles. For instance, when in time, enrolled patients have incrementally higher baseline-risk for adverse outcome at admission, (due to, for instance, stricter admission criteria), but individual treatment outcomes during admission improve over time. A mechanism that has been termed Simpsons Paradox [32]. The first two mechanisms were prevented by the cluster design of the study, in which all patients underwent the same cluster-intervention from the time of admission, precluding a distorted exposure-outcome relation. Yet, the third mechanism for ecological fallacy–differences in individual baseline and longitudinal risks—could have occurred.

For this analysis, available clinical cultures were used as endpoint determinant. The inclusion criterion of having a first negative enteric or respiratory culture ascertained acquisition of colonization with Gram-negative antibiotic resistant bacteria. The intervention is still ecological, but the analysis aims to provide individual risk estimates for colonization in a ward where antibiotic rotation is applied.

The restricted availability of these cultures prompted us to assess the presence and size of detection bias, which could have resulted from differential inclusion between intervention arms, due to the absence of individual randomization. We found no indication that baseline demographics or confounders were different between interventions, and adjustment for this in regression analysis did not change effect estimates. Alternatively, there could be a direct effect of the intervention on diagnostic culture practices. We used the source population (all included patients with and without cultures) to perform two additional analyses to assess inclusion bias or an intervention effect on culture rates: 1) Assessing the differences between interventions of the probability of a culture being taken by regression analysis, and 2) a sensitivity analysis to contextualize the size of the potential intervention-effect on detection bias and outcome.

The probability of having a culture taken was lower during the mixing period, but there was no evidence that this was due to differences in patient characteristics, nor were there differences in intervention effects after adjusting for confounders in the primary analysis. Sensitivity analysis yielded that detection bias would have needed to have caused a relative decrease in incidence of ARB acquisition with 29.2% (absolute 2.8%) to achieve statistical significance of the primary analysis. Or, in other words, in the uncultured patients in the mixing population that should have been cultured, based on our model, endpoint incidence would have to have been 16.6%, compared to 7.3% in the included mixing population, to achieve statistical significance. We consider this unlikely and therefore conclude that detection bias did not substantially reduce the validity of our analysis.

Naturally, generalizability of our findings is shifted towards patients with a clinical culture taken, and thus patients with a relatively long stay in ICU. In fact, despite generally a low threshold for collecting clinical cultures in ICU patients in the participating ICUs, excluded patients were mostly short-stay patients, discharged after a median 2 days of admission. Furthermore, results would not be generalizable to ICUs with higher endemic prevalence of antibiotic resistant bacteria, where the antibiotics that were used in this study cannot be used for empiric therapy. These results however, are generalizable to most European ICUs, and any non-European ICU with similar technical capacities, staffing and resistance prevalence.

## Conclusions

Our findings do not support superiority of effects of cycling over mixing or vice versa on acquisition of ARB in the participating ICUs. Based on current scientific evidence, including our study, antibiotic rotation should, therefore, not be recommended as standard care [13–27]. This study, however, rotated beta-lactam antibiotics exclusively, with fixed and pre-defined rotation schedules of per-patent rotation and 6-week periods. There are many variations of antibiotic rotation and effectiveness of some scheme is not excluded [33]. Resistance acquisition under antibiotic rotation strategies has layered complexity from the microbiome, infection control measures, and collateral sensitivity [34]. Further research is needed, and future clinical studies will benefit from a multi-disciplinary approach by including basic sciences and mathematical modeling. Ultimately the goal should be to provide tailor-made algorithms to guide ICU antibiotic policies, in order to optimize resistance selective pressure and patient safety.

## Supporting information

S1 Checklist CONSORT 2010 checklist of information to include when reporting a randomised trial*. (DOC)

S1 Appendix Appendix Tables 1–5. (DOCX)

S1 Protocol Protocol SATURN ICU trial. (DOC)

## Abbreviations

ARB: Antibiotic resistant bacteria
ICU: Intensive Care Unit
ASP: Antibiotic stewardship programs
DDD: Defined Daily Dose
aOR: Adjusted odds-ratio
