# Supplementary material for: The effects of antibiotic cycling and mixing on acquisition of antibiotic resistant bacteria in the ICU: A post-hoc individual patient analysis of a prospective cluster-randomized crossover study
Source: PLoS One. 2022 May 3;17(5):e0265720. doi: 10.1371/journal.pone.0265720 (PMC9064081; doi:10.1371/journal.pone.0265720)
Supplement: S1 Appendix — (DOCX) [file pone.0265720.s002.docx]

Appendix table 1 Material categories of all clinical cultures

| Material type | Cycling N (%) | Mixing N (%) |
| --- | --- | --- |
| Total | 9,245 | 7,209 |
| Respiratory | 3,060 (33.1) | 2,660 (36.9) |
| Blood or intravascular | 2,805 (30.3) | 2,112(29.3) |
| Enteric | 1,310 (14.2) | 701 (9.7) |
| Urine | 979 (10.6) | 999 (13.9) |
| Wound | 183 (1.98) | 125 (1.73) |
| Abdominal | 88 (0.95) | 51 (0.71) |
| Intracerebral | 69 (0.75) | 128 (1.78) |
| Other | 751 (8.1) | 433 (6.0) |

Appendix table 2 Identification of Gram-negative endpoint species*.

| Microorganism | Cycling N (%) | Mixing N (%) |
| --- | --- | --- |
| Total | 669 | 631 |
| *Escherichia coli* | 199 (28.6) | 172 (27.3) |
| *Pseudomonas aeruginosa* | 170 (25.4) | 106 (16.8) |
| Klebsiella species | 111 (16.6) | 133 (21.1) |
| Enterobacter spp. | 61 (9.1) | 84 (13.3) |
| Proteus spp. | 47 (7.0) | 27 (4.3) |
| Serratia spp. | 36 (5.4) | 32 (5.1) |
| *Acinetobacter species* | 15 (2.2) | 28 (4.4) |
| Citrobacter spp. | 12 (1.8) | 21 (3.3) |
| Morganella spp. | 10 (1.5) | 21 (3.3) |
| *Hafnia alvei* | 7 (1.0) | 1 (0.2) |
| Providencia spp. | 4 (0.6) | 2 (0.3) |
| Salmonella spp. | 4 (0.6) | 1 (0.2) |
| Raoultella spp. | 1 (0.1) | 3 (0.5) |

* Enterobacterales species, *Pseudomonas aeruginosa*, Acinetobacter species

Appendix table 3 Modelling carryover effects

| Variable | Adjusted model^a^ | p-value | Adjusted model with carryover interaction term^a^ | p-value |
| --- | --- | --- | --- | --- |
| Model fit change *ΔAIC^b^* |  |  | -4 (59.69 – 596.36) | *0.85* |
| Carry over model estimates | Hazard *ratio^c^*  (95% CI) |  | Adjusted hazard *ratio^c^*  (95% CI) |  |
| *Intervention^d^* | 0.62 (0.38 – 1.00) | *0.05* | 0.66 (0.35-1.28) | *0.22* |
| Intervention *sequence^e^* |  |  | 0.86 (0.40-1.85) | *0.69* |
| Interaction term (Intervention*sequence)*^f^* |  |  | 0.91 (0.33-2.50) | *0.85* |
| Age | 1.01 (0.996 – 1.022) | 0.17 | 1.01 (0.996 - 1.022) | *0.18* |
| Gender | 1.40 (0.86 – 2.28) | 0.18 | 1.39 (0.85-2.26) | *0.19* |
| Previous admission | 0.66 (0.41 – 1.07) | 0.09 | 0.66 (0.40-1.07) | *0.09* |
| Referral origin*^g^* | 1.24 (0.76 – 2.03) | 0.40 | 1.21 (0.74-2.00) | *0.45* |
| Length of stay | 1.07 (1.06 – 1.08) | <0.01 | 1.07 (1.06-1.08) | *<0.01* |
| Deceased during admission | 2.10 (1.21 – 3.63) | <0.01 | 2.10 (1.21-3.65) | *<0.01* |

*^a^* Interaction term of intervention and sequence of intervention (cycling-mixing or mixing-cycling)

*^b^* Difference after adding interaction term to model, tested with ANOVA

*^c^* Exponent of model coefficient

^d^ Cycling as reference

*^e^* Cycling then mixing as reference

*^f^* Interaction term between intervention and sequence

*^g^* Community versus hospital

Appendix table 4 Antibiotic consumption per 100 admission days

|  | Cycling | Mixing | p-value |
| --- | --- | --- | --- |
| Carbapenems | 21.98 | 19.25 | 0.07 |
| 3^rd^/4^th^ generation cephalosporins | 14.48 | 17.18 | 0.84 |
| Broad-spectrum penicillins w/ betalactamase-inhibitor | 13.89 | 13.11 | 0.15 |
| Piperacillin-tazobactam | 9.61 | 9.45 | 0.17 |
| Fluoroquinolones | 9.06 | 10.37 | 0.89 |
| Aminoglycosides | 2.55 | 2.50 | 0.15 |
| Cotrimoxazole | 1.77 | 2.67 | 0.53 |

Appendix table 5 Mixed effects logistic regression odds ratios for chance of having a clinical culture taken

| **Analysis type** | **Variable** | **Mixing:cycling odds ratio** | **Confidence interval**  **(2.5%-97.5%)** | **p-value** |
| --- | --- | --- | --- | --- |
| **Unadjusted *model ^a,b^*** | **Intervention ^c^** | 0.83 | 0.76 till 0.91 | <0.01 |
| **Adjusted *model ^d^*** | **Intervention** | 0.84 | 0.76 till 0.93 | <0.01 |
|  | **Age** | 1.00 | 1.00 till 1.01 | <0.01 |
|  | **Gender** | 0.97 | 0.88 till 1.08 | 0.59 |
|  | **Length of stay** | 1.27 | 1.25 till 1.30 | <0.01 |
|  | **Previous admission** | 2.10 | 1.88 till 2.34 | <0.01 |
|  | **Origin of referral** | 0.99 | 0.88 till 1.11 | 0.82 |

*^a^* All admissions during cycling and mixing interventions

*^b^* Adjusted variables: Random effect: Hospital

*^c^* Mixing is the reference

*^d^* Adjusted variables: Age, gender, length of stay, previous admission, community or hospital referral, random effect: Hospital
