# Supplementary material for: The effects of antibiotic cycling and mixing on acquisition of antibiotic resistant bacteria in the ICU: A post-hoc individual patient analysis of a prospective cluster-randomized crossover study
Source: PLoS One. 2022 May 3;17(5):e0265720. doi: 10.1371/journal.pone.0265720 (PMC9064081; doi:10.1371/journal.pone.0265720)
Supplement: S1 Protocol — (DOC) [file pone.0265720.s003.doc]

Protocol SATURN ICU Trial

Impact of **S**pecific **A**ntibiotic **T**herapies on the prevalence of h**U**man host **R**esista**N**t bacteria

June 2010

**Clinical trial registration:** TBD

**Version: 29 June 2010**

**Funding source:** European Commission, DG Research

**EU Project ID:** FP7-HEALTH-2009-SINGLE STAGE - N°241796

**Planned start of patient enrolment:**  January 2011

Impact of **S**pecific **A**ntibiotic **T**herapies on the prevalence of h**U**man host **R**esista**N**t bacteria

| Short title WP2 | SATURN-ICU Trial |
| --- | --- |
| Date | January 19 2010 |
| Coordinating investigator | P.J. van Duijn  M.D., Ph.D. Canidate  Julius Center for Health Sciences and Primary Care  University Medical Center Utrecht  Heidelberglaan 100  3584 CX Utrecht, The Netherlands  Email: [pduijn3@umcutrecht.nl](mailto:pduijn3@umcutrecht.nl) |
| Principal Inverstigator | Prof M.J.M. Bonten  M.D., Ph.D.  Professor of Molecular Epidemiology of Infectious Diseases  Julius Center for Health Sciences and Primary Care  University Medical Center Utrecht  Heidelberglaan 100  3584 CX Utrecht, The Netherlands  Phone: +31 30 2507394  Secretary: +31 30 2523741  Email: [mbonten@umcutrecht.nl](mailto:mbonten@umcutrecht.nl) |
| **SATURN Coordinator:** | Stephan Harbarth, MD, MS  Infection Control Program  University of Geneva Hospitals and Medical Faculty  4 Rue Gabrielle Perret-Gentil,  1211 Geneva 14 - Switzerland  Phone: (+41) 22 372 3357  Fax: (+41) 22 372 3987  Email: [stephan.harbarth@hcuge.ch](mailto:stephan.harbarth@hcuge.ch) |

**Protocol Signature Sheet**

| Name | Date | Signature |
| --- | --- | --- |
| Head of Department:  M.J.M. Bonten, MD, PhD  Professor of Molecular Epidemiology of Infectious Diseases |  |  |
| Coordinating Investigator:  P.J. van Duijn, MD  PhD Student |  |  |
| Principal Site Investigator  .................................. |  |  |

**Table of Contents**

**Study synopsis**

**1. Introduction………………………………………………………….2**

**SATURN consortium**

**ICU trial rationale**

**ICU Trial Outline**

**SATURN study partners involved**

**Impact**

**2. Objectives…..………………………………………………………..5**

**Primary endpoint**

**Secondary endpoints**

**Timeline**

**3. Methods………………………………………………………………6**

**3.1 Interventions………………………………………………….6**

**Phase 1 – Baseline measurements**

**Phase 2 - Intervention**

**Mixing and Cycling antibiotics**

**Selection of antibiotic classes**

**Selection of pathogens**

**3.2 Data collection………………………………………………..9**

**Patient-data**

**Demographics on admission**

**Antibiotic usage data**

**Microbiology data**

**Data from routine clinical cultures**

**ICU parameters**

**ICU characteristics**

**Selection of eligible ICUs**

**3.3 Study population………………………………………….….13**

**Sample size calculations**

**3.4 Statistical analysis plan……………………………………..15**

**Missing data**

**Changes to the statistical analysis plan**

**4. Explanatory list of concepts……………………………………….16**

**Nurse-to-patient staffing ratio**

**Hand hygiene compliance**

**5. Financial compensation…………………………………………....17**

**6. Site withdrawal or Discontinuation of Trial……………………..18**

**7. Safety Reporting……………………………………………………..19**

**8. Ethical considerations………………………………………………19**

**Regulation statement**

**Recruitment and consent**

**Informed Consent Waiver**

**Benefits and risks assessment**

**Patient burden**

**Patient risk**

**Compensation for injury**

**9. Administrative aspects and publication………………………….22**

**Handling and storage of data and documents**

**Confidentiality**

**Amendments**

**Annual progress report**

**Public disclosure and publication policy**

**10. References…………………………………………………………..24**

**List of abbreviations**

ADL Activities of Daily Life

AMRB AntiMicrobial Resistant Bacteria

ASP Antibiotic Stewardship Programme

ATS American Thoracic Society

AU Antwerp University

BSI Blood Stream Infection

CRT Cluster Randomized Trial

ESBL Extended Spectrum Beta-lactamases

EU European Union

GNARB Gram-Negative Antimicrobial Resistant Bacteria

HCW Health Care Worker

HH Hand Hygiene

ICU Intensive Care Unit

IDSA Infectious Diseases Society of America

IDIBAPS l’Institut d’Investigacions Biomèdiques August Pi i Sunyer

IRB Institutional Review Board

LOS Length Of Stay

MRSA Methicilline Resistant Staphylococcus Aureus

RN Research Nurse

UMCU University Medical Center Utrecht

VRE Vancomycin Resistant Enterococcus

WHO World Health Organisation
WP Work Package

**Study synopsis**

**In a cluster-randomized cross-over multi-center trial we will determine the effects of two antibiotic strategies (cycling and mixing) on the prevalence of antimicrobial resistant bacteria (AMRB) in 10 intensive care units (ICU). Cycling implies the temporary use (i.e., 1,5 months) of an antibiotic of a certain class as preferred treatment for Gram-negative infections. During one cycle, antibiotic exposure will be maximally homogeneous as different patients will be treated with the same class of antibiotics. After this period, antibiotics from a different class of antibiotics will be preferentially used in order to change antibiotic selective pressure. During mixing, the preferred class of antibiotics will change per consecutively treated patient. In this strategy antibiotic use will be maximally heterogeneous. The total cycling and the mixing intervention-period will last for nine months. The primary endpoint of the study will be the difference in the average prevalence of AMRB in the ICU during both strategies. This endpoint will be determined through monthly point-prevalence surveys of respiratory tract and rectal samples from all patients present in the ICU at that time point. Secundary endpoints include acquisition rates of respiratory tract carriage with AMRB, bacteremia rates with AMRB and appropriateness of treatment of bacteremia.**

**This study will quantify the ecological effects of antibiotic cycling and mixing on the prevalence of AMRB in ICU.**

**1. Introduction**

**SATURN Consortium**

**Antibiotic resistance has exponentially grown in the last ten years, especially within Intensive Care Units (ICU). The spread and increase of antimicrobial resistance bacteria (AMRB) has turned the potential of this phenomenon from a concern for policymakers into a practical problem affecting physicians from medical and surgical specialties alike.** The problem of increasing antimicrobial resistance is even more daunting due to the lack of newly developed antimicrobials in recent years. Moreover, the future outlook is equally dismal. In a recent report ECDC and EMA described that - worldwide - there are only two new antimicrobial agents with new or possibly new modes of operation in clinical or pre-clinical development phases ^1^.

**The SATURN Consortium aims to tackle the problem of antimicrobial resistance (AMR) by studying the emergence of resistance in four clinical trials combined with a pharmacodynamic and microbiological workpackage providing additional diagnostics and analysis on the clinical data.**

Many results drawn from previous studies of the effect of antibiotic use on emergence, selection and spread of antimicrobial resistance have lacked a holistic view combining all aspects into one study. The SATURN project aims to study the impact of antibiotic exposure on AMR with a multidisciplinary approach that bridges molecular, epidemiological, clinical and pharmacological research. Two types of clinical studies will be conducted: First, a randomised trial will be performed to resolve an issue of high controversy (antibiotic cycling versus mixing). Second, 3 observational studies will be conducted to rigorously study issues surrounding the effect of antibiotic use on AMR that are not easily assessable through randomised trials. These clinical studies will serve as a platform to 2 complementary workpackages (microbiology and pharmacology) that will perform important investigations relevant to this call. The work package focusing on molecular studies will generate new

evidence on the changes effected by antibiotic therapy on commensal organisms or opportunistic pathogens in the oropharyngeal, nasal and gastro-intestinal flora. Additionally, we will study different AMR mechanisms and the dissemination of successful clones of fluoroquinolone-resistant, carbapenem-resistant or extended-spectrum beta-lactamase harbouring Gram-negative bacteria, MRSA and fluoroquinolone-resistant viridans streptococci. The purpose of the pharmacodynamic study is to model the relationships between antibiotic exposure and AMR emergence over time for various classes of agents. In summary, the overarching rationale of SATURN is to improve methodological standards and conduct research that will help to better understand the impact of antibiotic use on acquisition, selection and transmission of AMR in different environments, by combining analyses of molecular, individual patient-level and ecologic data. The anticipated results may guide clinical and policy decisions to ultimately reduce the burden of AMR in Europe.

This protocol describes the rationale, design and procedures of the intervention-trial (WP2) conducted in intensive care units (ICUs), which is a clinical study on the impact of two antibiotic prescription strategies (mixing and cycling) on AMRB prevalence in a heteregeneous group of ICUs.

The microbiological data from clinical samples, combined with epidemiological and pharmacological data from clinical trials, will be analysed in the Bacterial Genetics and Functional Studies (WP1) and the Pharmacodynamic Study (WP6). Work Package 7 (WP 7.1 and 7.2) will be responsible for Project Management and Dissemination of scientific data and their concomitant preventative and therapeutic recommendations.

**ICU trial rationale**

Infections caused by AMRB adversely affect ICU patient treatment efficacy, and have been associated with prolonged length of stay (LOS), increased use of health care resources and costs^2, 3^. The recent rise in AMRB colonized and infected patients reduces treatment options for Intensive Care physicians. Because of its specific microbiological ecology, high antimicrobial pressure, vulnerable patient population, and multiple contacts between health care workers and patients, ICUs are considered hot-spots of AMRB prevalence.

To reduce (or modulate) antibiotic pressure, several antibiotic prescription strategies have been proposed. Most Antimicrobial Stewardship Programs (ASPs) aim to reduce overall antibiotic use, either by optimizing de-escalation and/or by improving microbial-specific antibiotic prescription^4^, and these strategies are nowadays widely recommended in local and national guidelines.

The effects of two other strategies are less well studied: antibiotic mixing versus antibiotic cycling. **Antibiotic cycling** is a strategy in which antibiotic groups with the same spectrum of activity are temporarilly cycled in a serial manner, in order to intermittently change the selective antibiotic pressure. During one period, all patients in a ward needing empiric antibiotics receive antibiotics from a single class. Usually after a period of one to six months the preferential antibiotic group is changed or “Cycled”. During each cycle period there is homogeneity of antibiotic selective pressure. **Antibiotic mixing** aims to create maximum heterogeneity of antibiotic selective pressure, by alternating several classes of broad-spectrum antibiotics in consecutive patients without any systematic pre-defined schedule.

The cycling strategy assumes that periodically changing homogeneous antibiotic pressure, reduces overall selective pressure on microbial ecology. Hypothetically, any growth and transmission of resistant bacteria will be interrupted by cycling to another antibiotic class; either due to direct effects of the new antibiotic class (as long as the resistant strains are still susceptible to the new class) and/or due to a change in selective pressure. Due to fitness-cost of acquiring antibiotic resistance AMRB will be less prone to survive in a different microbial milieu.

Interpretation of data available on both strategies, though, has been hampered by methodological flaws in study design and data analysis, such as the lack of adjustment for important confounders like severity of disease, infection control measures and antibiotic use, and the absence of knowledge on the importance of exogenous routes of AMRB acquisition ^5^. The currently available evidence is not sufficient to recommend either cycling or mixing of antibiotics in ICUs ^6-17^. Clearly, there is a need to methodologically improve previously generated evidence in this area and provide high-quality data to clinicians and policy makers.

**ICU Trial Outline**

It has been hypothesised that scheduled changes in the predominant use of certain antibiotics in the ICU setting, also called ’antibiotic cycling’, will reduce overall AMRB, but positive findings were not confirmed in other studies [reviewed by Nijssen et al.]. Importantly, confounding by unmeasured variables was not excluded in any of these studies, and neither were appropriate statistical tests used for capturing non-linear dynamics. On the contrary to the results of some clinical studies, there is theoretical evidence that such a strategy would increase AMRB, and that it would be better to change antibiotic classes per patient (’antibiotic mixing’). Hitherto, no well-conducted prospective study has demonstrated that antibiotic cycling or mixing affect the development and spread of ARB in the ICU setting.

The main hypothesis to be tested is that antibiotic cycling and antibiotic mixing differently affect the bacterial ecology, defined as the carriage rates of AMRB in the respiratory and digestive tract of ICU patients. It is further hypothesised that antibiotic cycling vs. mixing differently affect the rates of endogenous and exogenous acquisition of AMRB by ICU-patients. Based on the existing evidence, both from experimental studies as well as from theoretical models, it is still uncertain which of the two strategies is most likely to be associated with the most beneficial effect on AMRB in ICUs.

Important confounders for these endpoints, i.e. adherence to hygienic measures and colonisation pressure, will also be determined.

Since cycling and mixing intend to have an ecological (unit-wide) effect, both interventions should be studied when implemented as a unit-wide intervention. Moreover, since AMRB might be transferred from patient to patient, patient-dependency should be considered when analyzing the effects of both interventions. Finally, it is preferable to investigate such strategies in multiple wards, as this will increase generalizibility of findings and will generate more power to identify relevant outcome differences. Yet, as different units are – by definition – different in their risk profiles for the study endpoints, one would either need many different wards (each using a single intervention) or apply a cross-over design in which each unit is subjected to both interventions (with the order randomized). All these aspects can be incorporated in a multi-center cluster-randomized cross-over study.

**SATURN study partners involved**

Data on individual and aggregated antibiotic consumption and carriage or infection with AMRB will be provided to WP6 for pharmacokinetic modelling. AMRB isolates will be provided to different partners for in-depth microbiologial analyses.

**Impact**

This study will evaluate antimicrobial strategies that are universally and directly deployable. The outcome results will, therefore, be immediately applicable for ICUs in Europe, as well as in other continents.

**2. Objectives**

**Primary endpoint**

The primary endpoint of this trial is the difference in unit-wide AMRB prevalence (in %), expressed as the difference in average point-prevalence in the participating 10 ICUs of antibiotic-resistant *Enterobactericae*, *Acinetobacter spp.* and *Pseudomonas aeruginosa*,stratified by intervention arms (antibiotic cycling versus mixing).

**Secondary endpoints**

Secondary outcome measures are:

- AMRB acquisition incidence, measured as status conversion from non-colonized to colonized during admission at ICU per 100 patients.
- ICU-acquired bacteraemia rate with AMRB (expressed as the rate of ICU-acquired bacteraemia per 1000 patient-days)
- Overall length of ICU-stay hospital-stay and percentage of in-hospital mortality of the total admitted ICU-population.
- Effectiveness of empirical treatment of ICU-acquired bacteraemia (expressed as proportion of bacteraemia for which appropriate antibiotics are administered within 24 hours after obtaining bloodcultures, with appropriate defined as an antibiotic for which the pathogen has in vitro susceptibility and is given for more than 5 days)

**Timeline**

|  | DELIVERY (months) | DEADLINE |
| --- | --- | --- |
| START SATURN | 0 | Feb 2010 |
| STUDY PROTOCOL | 12 | Jan 2011 |
| SITE SELECTION | 12 | Jan 2011 |
| LAB MANUAL | 12 | Jan 2011 |
| PHASE 1 - BASELINE DATA | 20 | Sep 2011 |
| PHASE 2 - INTERVENTION DATA 1 | 30 | Jul 2012 |
| PHASE 2 - INTERVENTION DATA 2 | 40 | May 2013 |
| ANALYSIS & REPORTING | 51 | Apr 2014 |

**3. Methods**

**3.1 Interventions**

The ICU trial is an interventional cluster-randomized cross-over multi-center trial.

In two periods of nine months, ICUs will implement an Antibiotic Stewardship Programme to execute both strategies.


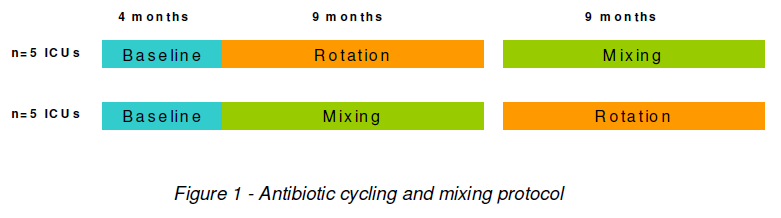


**Phase 1 – Baseline measurements**

After ICU-site selection, baseline measurements will be performed during 4 months in all sites. Treatment and isolation precautions for outbreak-management will be implemented according to standard-care without any interventions. During this Phase 1, primary and secondary endpoint data will be collected.

**Phase 2 - Intervention**

After the base-line period, all ten ICUs are randomized, and allocated to the “Cycling” or “Mixing” antibiotics-group for a period of nine-months. After a one-month wash-out/wash-in period, both interventions will be crossed-over in all sites (mixing to cycling or vice versa). For the clusters in the Cycling group, antibiotic classes will be cycled every 1,5 months. In both groups, endpoint measurements will be obtained as in Phase 1. The primary endpoint will be determined through monthly point-prevalence surveys of microbiological screening for colonization with Gram-negative bacteria in the respiratory (tracheal aspirate or oropharyngeal swab) and rectum (perineal swab). Furthermore, all results from clinical microbiological cultures obtained at the discretion of treating physicians will be collected. Finally, there will be on-site observations of Hand Hygiene Compliance and audits of antibiotic prescribing.

As the hypothesized effect of the intervention is on an ecological scale, all patients in the ICU will be included.

**Mixing and Cycling antibiotics**

During the intervention phase of the trial, antibiotic prescription for suspected Gram-negative infections will be protocolized. The treating physician will initiate treatment based on normal standard of care decision making. There are no other changes in patient-care regarding treatment, de-escalation, diagnostics, patient-informing or changes in infection prevention.

*Mixing*

The “Mixing”-protocol inlcudes the prescription of different antibiotic classes for each new consecutive antimicrobial treatment. Each consecutive patient needing empiric antimicrobial treatment (at the discretion of treating physicians) and needing coverage of Gram-negative bacteria will receive antibiotics from a different class. In this way, patients are assigned to a specific antibiotic class and will continue to receive that antibiotic for the duration of their treatment, unless treatment with a different class is preferred (at the discretion of treating physicians). The order of antibiotic groups is predefined and will remain unchanged for all consecutive patients in one cluster.

*Cycling*

The “Cycling”-protocol includes the prescription of a single class of antibiotics for all consecutive patients needing empiric antimicrobial treatment (at the discretion of treating physicians) and needing coverage of Gram-negative bacteria during a predefined period. In this way, all patients are assigned to a specific antibiotic class and will continue to receive that antibiotic class for the duration of their entire treatment, unless treatment with a different class is preferred (at the discretion of treating physicians). After this period the preferred antibiotic class for treatment of Gram-negative infections is changed. Antibiotic classes will be cycled every 1,5 months. The order of antibiotic groups is predefined and will remain unchanged in each unit.

*Deviations from protocol*

The protocol can be overruled by the treating physician at any time, before or during treatment, for reasons of antibiotic hypersensitivity, non-matching susceptibility-pattern, antibiotic de-escalation or any other reason considered necessary to improve patient-care. The target adherence level to protocol is 80%, and will be measured from aggregated data on antibiotic consumption combined with either complete individual data, or a representative sample size of patient-specific antibiotic prescription data regarding protocol compliance.

**Selection of antibiotic classes**

Three groups of antibiotics were preliminary selected for usage in the mixing and cycling protocols. These antibiotics represent among the most frequently used agents in ICU patients worldwide, and are in agreement with international guideline recommendations (such as the guidelines from ATS, IDSA and ESICM) ^18-20^.

These antibiotics are:

- a 3rd- (or 4th-) generation cephalosporin,
- a carbapenem
- piperacillin-tazobactam

Fluoroquinolones are not included as an antibiotic class for cycling or mixing because of international existing levels of antibiotic resistance of some pathogens and because of the reported association with rapid selection of antibiotic resistance during extensive exposure^5, 21^. Aminoglycosides are (almost) never used as single treatment and their use in combination treatment with any of the selected antibiotics will not be protocolized, but left to the discretion of the responsible physicians. Furthermore, antibiotic treatment for Gram-positive infections will not be protocolized.

**Selection of pathogens**

Selected bacteria are:

- ESBL-producing Enterobacteriacae
- Pip/tazo-resistant Enterobacteriacae, *P. aeruginosa* and Acinetobacter species
- Carbapenem-resistant Enterobacteriacae, *P. aeruginosa* and Acinetobacter species

**3.2 Data colletion**

**Patient-data**

Data are collected from the digital patient-management file, regarding patient-demographics, patient-risk factors for colonization (i.e., previous colonization, previous admission, prior location before admission), illness severity-scores.

**Demographics on admission**

The following demographic data will be derived from all patients on admission to the ICU:

- Sex
- Age
- Date of admission and date of discharge from the ICU
- Colonization with GN AMRB on admission
- Location prior to ICU and hospital admission
- Reason for ICU admission
- Extraction of severity of disease classification-scores (APACHE-III, SAPS II or SAPS III score) from digital patient-management systems.

**Antibiotic usage data**

Antibiotic data will be retrieved in two forms: Aggregated data and individual usage. Total aggregated antibiotic use is used to describe the overall antibiotic use during mixing and cycling, in order to quantify changes in exposure. In addition, individual antibiotic data will be collected from a random sample of patients in order to quantify protocol adherence during mixing and cycling. Finally, appropriate treatment of ICU-acquired Blood Stream Infections (BSIs) will be assessed using individual antibiotic data and antibiotic susceptibility of isolated pathogens.

Total antibiotic use during intervention periods will be collected on a unit-wide level; i.e., the total number of grams per period (which will be subdivided in grams per month/week/day). Preferably data will be directly extracted from a digital database and sent via e-mail to UMCU in a universal format. Otherwise data will be extracted manually by the Research nurse (RN).

**Microbiology data**

The primary study endpoint will be determined through monthly point-prevalence measurements of samples from the respiratory tract (either endotracheal aspirates or oropharyngeal swabs) and samples from the perineum (perineal swabs). These point-prevalence swabs will be obtained from all patients present in the ICU at the time of the point-prevalence survey. Dates and times for obtaining monthly point prevalence data will be assigned per participating ICU.

Because of logistics, swabs will be taken during office-hours. Swabs will be frozen at -70ºC and stored at the subcontractors hospital and shipped in batches to UMCU (*Appendix 12.2*). For the transport of isolates, a specialized courrier will be used that has proven experience in this type of shipments. Swabs will be screened at the UMCU Microbiology Laboratory for presence of selected pathogens and resistance typing.

Testing of ESBL-harbouring *Enterobactericae* is done using chromogenic media, and for *P. aeruginosa* and *Acinetobacter spp.*, using antibiotic-containing selective media followed by species identification on a semi-automated system (Vitek 2, bioMérieux).

**Data from routine clinical cultures**

All non-study related microbiological culture results obtained during ICU stay will be collected as well. These data will be sent periodically – in digital format - to the UMCU. Data will include type of sample, culture result and antibiotic sensitivity pattern.

**ICU-parameters**

Occupancy rates, use of invasive devices, parenteral nutrition and patient-specific antibiotic use will be determined through monthly point-prevalence surveys. Dates and times for obtaining monthly point prevalence data will be assigned per participating ICU.

The following information will be collected:

**Number of patients with**:

- Mechanical ventilation
- Intracranial monitor
- Tracheostomy
- Intravascular catheters (e.g. central venous catheter, arterial catheter, haemodialysis catheter)
- Parenteral nutrition
- Isolation precautions

**Number of beds:**

- Available
- Occupied

**Number of nurses working in the unit**

**ICU Characteristics**

ICU characteristics will be determined at the beginning of the baseline period (month 1), and every 6 months afterwards. Data collected will consist of:

- Name of physician director of ICU and primary specialty (changes herein will be recorded)
- Name of nurse director (manager) of ICU (changes herein will be recorded)
- Previous or current hand hygiene promotion, infection prevention program or any investigational infection prevention or treatment programme in the ICU
- Total amount of beds and ventilators available
- Antibiotic policies and guidelines in place
- AMRB situation in the preceding year with indicator organisms and total AB usage (only collected before baseline).

**Selection of eligible ICUs**

ICUs will be selected on their capacity to generate high-quality data. This includes a suitable environment for clinical and microbiological data collection and performing on-site observations.

An ICU-ward is defined as a separate ward, physically distinguished from other, non-ICU-wards. As part of EU legislation, putative subcontractors will be given the opportunity to apply for a position as a subcontractor in the WP2 ICU trial. ICUs will be informed through the websites of the Julius Center UMC Utrecht, European Society of Clinical Microbiology and Infectious Diseases (ESCMID) and the European Society of Intensive Care Medicine (ESICM)^19, 22^. Through a procedure legislatively outlined by the EU, candidate-institutions will be allowed to submit their application. After initial selection by the WP2 Principal and Coordinating Investigator, a selection of ICUs will undergo an on-site audit. From these ICUs, ten units will be selected for trial participation. If possible, ICUs will be included in a ratio of 5 older member states: 5 new member states.

ICUs can be included if:

- There are at least 8 beds, with an average bed-occupancy of 80%; all of which have capacity for mechanical ventilation.
- The ICU can adhere to the selected antibiotics for empiric treatment of infections.
- There is an operational digital patient-information system, from which data can be extracted and delivered in a pre-defined format. Specifically an automated process for digital data-collection regarding microbiological culture-results (from swabs and bacteraemias), antibiotic prescription and patient demographics and illness severity-scores.
- Colonization with ESBL or resistance for any of the antibiotic groups is endemic, with proportions of ICU-acquired bacteraemias used as a proxy. Therefore, we prefer proportions of AMRB infection in the period 2008-2009 to be:

ESBL resistance among GNB 1 to 10%

Piperacillin/Tazobactam among GNB 1 to10%

Carbapenem resistance among Klebsiella Pneumoniae less than 5%

- Have the ability of at least one dedicated Infection Control HCW available for 0,2fte, for patient monitoring, compliance monitoring and instruction of HCWs regarding interventions. In the following this person will be called “Research-Nurse” or “RN”.
- Can store screening-cultures at -70ºC
- Can facilitate transport through a UMCU courier.
- There is written approval for the study from the institution’s IRB with a waiver for patient informed consent.
- A signature page is signed by the daily management of the candidate-ICU by both ICU physician-director and the ICU nursing-director and presented to the UMCU, indicating willingness to enroll the candidate-ICU in the study.

We will use the following exclusion criteria:

- ICUs planning to introduce, during the SATURN trial period, any major diagnostic- or intervention program that will affect AMRB ecology*
- Burn units; due to the specific nature of the care provided and the patients admitted.
- Cardiothoracic surgery units; because of the expected small number of patients admitted for three days or more.
- Paediatric and neonatal ICUs.

*E.g.: Hand Hygiene Improvement Programs, Chlorhexidine body washings, SDD, SOD, or ICUs planning to enroll subjects in any other studies, testing investigational agents for the purpose of eradicating or preventing colonization with MRSA, VRE, ESBL or devices or practice management strategies that have colonization and/or infection with resistant organisms as an outcome. Any program, agent or research-trial that is already active in the ICU should not be halted or changed during the intervention period.

ICUs will be denoted as Medical, Surgical or Medical/Surgical-ICU according to the following descriptions:

- Medical ICU: ≥80% of admitted patients have medical conditions and have not undergone a surgical procedure during their hospital stay, excluding small, out-patient procedures.
- Surgical ICU: ≥80% of admitted patients have undergone a surgical procedure during their hospital stay (thoracic, cardiovascular, abdominal, orthopaedic, neurosurgical, transplant, urologic, and trauma surgery procedures may be included);
- Medical/surgical ICU: a roughly equivalent mixture of patients with medical conditions who have not undergone surgical procedures and patients who have undergone surgical procedures during their hospitalization (i.e., each group constitutes more than 20% and less than 80% of the total number of patients).

**3.3 Study population**

For the primary endpoint analysis (average of point-prevalence surveys) and the aggregated antibiotic data analyses all ICU-patients will be included. Of note, we aim to investigate the ecological (unit-wide) effects of both antibiotic regimens on antibiotic resistance, rather than their effects on antibiotic resistance in individual patients. Therefore, no patients will be excluded, and all patients present in the ICU at the day of the point-prevalence measurement will be included, regardless of their length of stay.

For the secondary endpoints (such as incidence of acquisition of colonization and bacteraemias, length of ICU-stay, appropriate treatment) only patients with an ICU-stay of >48 hours will be included. All patients will be included for assessing acquisition incidence, bacteraemia rate and length of stay. Data on appropriate treatment will be taken from patients with BSI.

The study will only address the effects of both regimens on the ICU-level, without follow-up of patients after ICU-discharge.

**Sample size calculations**

Power calculations for, and analysis of, controlled trials in which the unit of allocation is the individual patient, assume that observations on individuals are independent.

This is different for clustered trials, where observations on individuals within the same cluster may be correlated and are interdependent.

Since correlated responses do not contain as much information as independent responses, within-cluster correlation affects the power of a trial and the precision of the estimates of effect, and should, therefore, be taken into account. The extent to which a decrease in the within-cluster correlation increases the sample size required for a study (i.e. the design effect or inflation factor) depends on the average cluster size (m) and the intra-class correlation coefficient (ρ), and is given by:

*design effect* = 1+ (*m* −1) ρ

The *design effect* is the ratio of the variance of an estimator under cluster sampling to its variance under individual random sampling:

*ρ = σ^2^ _intra_ / σ^2^_intra_ + σ^2^_inter_*

Where *σ^2^_inter_* is the inter-cluster component of the variance and *σ^2^_intra_* is the intra-cluster component of the variance. If the design effect is 4, then a cluster trial will require four times the number of individuals required by a simple randomized trial of individuals. When m = 1, we have a simple randomized controlled trial with a design effect of 1. Similarly if there is no within-cluster correlation, the between-cluster variance will be 0, *ρ* = 0 and again the design effect will be 1.

In a recently performed cluster-randomized cross-over study in 13 Dutch ICUs the calculated intra-class correlation coefficient (ICC) was <0.0183^23^.

With an expected number of patients per cluster of 270 (18 point-prevalence measurements times 15 patients per ICU) and ICC= 0.01, estimated sample size without clustering taken into account = 400 patients (based on a perceived absolute difference of 10% with standard deviations of 5) and the estimated number of clusters can be calculated:

Number of Clusters Needed = ((1+ (m-1) *ρ*) * 400)/m = 6

Therefore, 10 ICUs (= 20 clusters) should be sufficient for the proposed study, even when there is evidence for clustered effects.

**3.4 Statistical analysis plan**

A number of approaches are possible for the analysis of cluster-randomized trials, the most common being generalized linear mixed (multilevel) models and generalized estimating equations (GEEs). These can, sometimes, give somewhat different results.

Before selecting the final method of analysis, we propose to use simulation studies to guide the final choice between these methods. Both multilevel models and GEEs are also appropriate for analyzing longitudinal data from the study designed to evaluate the effect of the different antibiotic strategies. Again, models will account for unit-level clustering and, if necessary, autocorrelation and pre-intervention trends, seasonal effects, and measured confounding factors. The final choice between these analytical methods will again be guided by simulation studies.

One of the secondary endpoints will be the determination of the relative importance of endogenous (i.e., patient-independent) and exogenous (i.e. patient-dependent) transmission during both interventions. For this, we will use all available microbiological data, which means from samples obtained for clinical reasons as well as those obtained as part of the point-prevalence studies. With this information, each patient can be categorized as being colonized or not with an ARB of interest for as many days as possible. This will be the input for recently developed mathematical models (based on Markov Chain methods) to quantify the different acquisition routes. Days with unknown colonization status (as cultures will not be obtained on every day) will also be used in these models.

**Missing data**

Study investigators and the SATURN staff will make every attempt to collect complete data from all subjects enrolled in the study. Should surveillance cultures be inadvertently lost, those data will be treated as missing at random. Relative to the primary analysis, all inferential analyses will be based on available data. Details of the procedures for addressing missing surveillance cultures will be provided in the Statistical Analysis Plan. For secondary descriptive analyses, considerations for addressing other types of missing data will be handled on a case-by-case basis.

**Changes to the statistical analysis plan**

Details of the analysis methods and changes in the analyses from those described in the protocol will be documented in the Statistical Analysis Plan prior to database lock. Those changes and the reasons for the changes will be described in detail in the final study report.

**Confounding factors**

The following parameters, which may act as confounders, will be determined prospectively.

- - Patient-specific illness severity scores (APACHEIII and SAPSII and III)
  - Overall antibiotic use in grams per period (day, week, study period) on an aggregate level
  - Compliance with Contact Precautions, Standard Precautions and adherence to Hand Hygiene Guidelines (in %)
  - Staffing ratio’s of HCWs involved in direct patient-care

**4. Explanatory list of concepts**

**Nurse-to-patient staffing ratio**

Staffing ratios and cohorting levels can be important confounders. As it is considered impossible to intervene in these variables, their values will be monitored prospectively in order to be included as covariates in the final analyses. Nurse-to-Patient Staffing Ratio’s will be calculated every month. The RN will collect data regarding the nurse-to-patient staffing ratio for a 24-hour period (08:00 to 08:00) once every month on dates distributed randomly over the baseline and intervention periods. The ratio will be calculated as the number of nurse hours in a 24-hour period per patient day^24^. For instance, a ratio of 24 nurse hours per patient day represents an average of a 1:1 nurse-to-patient ratio for the entire 24-hour period. The research-assistant will review work assignment records and record the number of nurse hours for nurses assigned to direct patient care (e.g., a nurse assigned to direct patient care for an 8-hour shift would contribute 8 nurse hours). Charge nurse assignments will be recorded in the same fashion as other nurses if the charge nurse had a direct patient care assignment. The number of nurse’s aide hours will be recorded, but will not be included in the nurse-to-patient staffing ratio. The total number of patients cared for in the ICU during the 24-hour period and the number of admissions will be recorded in order to calculate the number of patient days.

**Hand hygiene compliance**

The research-nurse will conduct bedside (i.e., in the patient room) observations of the opportunities for hand hygiene during patient care. The Research-Nurse will perform an average of 4 observations of 15-30 minutes each per month during both the baseline and intervention periods. Because contact between patients and HCW is most frequent during these hours, and for feasibility reasons, the RN will perform these observations during day and evening nursing shifts on weekdays, but not on weekends or at night. This methodology is based on the WHO Hand Hygiene campaign and has been successfully used before in the MOSAR ICU trial.

All patients in the ICU at the time of the observations will be eligible for monitoring. Selection of patients to monitor will occur randomly. If observations cannot be performed on a selected patient for logistic reasons or because a patient, a family member, or a HCW requests that observations not be performed, the next patient on the random list will be monitored.

HCWs will be informed at the beginning of the study that observations of the frequency of patient care practices (not defined further) will be performed. If required by the site IRB, patients and their families will also be informed. If asked, the research-assistant will be instructed to state that they are observing the frequency of various patient care practices for the study. No further information about the specific observations will be divulged.

Observations will be recorded anonymous for both patient and HCW.

Data will be gathered on a maximum of 4 HCW per session, for a maximum of 10 hand hygiene opportunities per HCW.

For each session, the monitor will record the:

- Date of the observation, start and stop time, bed space number and initials of the observer
- Use of hand hygiene for each opportunity (handrub, handwash, new gloves or missed)

The monitor will only observe whether hand hygiene was performed. The monitor will not make judgments about whether these practices were performed optimally. For example, the monitor will not record information about the duration of hand washing.

The research-nurse will be instructed not to give any feedback about the adequacy of the practices he or she observes to the hospital, the HCW or the ICU patients or their family members.

The observer will not engage in patient care unless an emergency occurs (e.g.: resuscitation).

Observations of hand hygiene will be according to the WHO “Clean Care is Safer Care” program. An extensive description of the Bedside Monitoring can be found in the Bedside Monitoring Protocol in the Manual of Operations and an article by Sax et al ^25^.

**5. Financial compensation**

The UMCU will compensate the department of the principal investigator for the value of 0.2 FTEs of a Research Nurse position.

Below is a putative description for personnel involved in the data-collection in the ICU, and their respective estimated time burden, which is based on our experience during the MOSAR ICU-trial.

| **Function** | **Tasks** | **Frequency** | **Time Burden** |
| --- | --- | --- | --- |
| Research Nurse | Point-prevalence data collection | monthly | 240 min/month |
|  | Point-prevalence swabs (collection and freezing) | monthly | 360 min/month |
|  | Antibiotic prescription data collection | Every 6-12 months | 340 min/month* |
|  | Culture-data collection | Every 6-21 months | 120 min/month* |
|  | Hand Hygiene Compliance observations |  | 120 min/month |
|  | Training of physicians and supervision of antibiotic-protocol | Weekly | 340 min/month |
|  | Miscellaneous** |  | 400 min/month |
|  |  | **Total** | 32 hours/ month |
| Principal Investigator | Supervision of Research Nurse, Quality Control of methodology and data-collection. |  |  |
| ICU Physician-Director | Supervision of protocol, data collection on ICU characteristics |  |  |

*Depending on type of ICU data-management (digital or manual)

**Correspondence with UMCU and other partners, training for HH compliance-observations, sending shipments of frozen swabs, contingency management etc. etc.

The compensation will be divided into roughly 3 terms and compensation for the next term will only proceed if data has been delivered for the previous term.

Definite failure to provide qualitative and quantitative data as described in this protocol will result in discontinuation of the ICU as subcontractor within the SATURN ICU-trial and the compensation received for data not delivered will be reclaimed by the Julius Center.

Patients will not be compensated for their participation in the trial.

**6. Site withdrawal or discontinuation of trial**

The ICU physician-director or the Site Investigator may withdraw his or her site from the study for any reason at any time. In case of an unexpected change in AMRB epidemiology in a certain ICU, creating a situation in which adherence to protocol can no longer be recommended the site will temporarily be withdrawn. If withdrawal exceeds >3 months the site will be excluded.

The WP2 investigators may discontinue the participation of a study site for the following reasons:

- The site does not follow the study protocol with respect to obtaining the surveillance cultures.
- The site does not implement the assigned strategy to satisfaction.
- The site does not collect the required patient information.
- The site is unable to produce the required data in a digital format suitable for analysis.
- During the trial the ICU enrolls subjects in a study administering an investigational agent, or diagnostic test performed, for the purpose of controlling, eradicating or preventing colonization with ESBL.
- The site’s IRB does not give a waiver for informed consent.

**Withdrawal of individual subjects**

Subjects can leave the study at any time for any reason if they wish to do so, without any consequences whatsoever.

**7. Safety reporting**

**Adverse events**

This protocol does not involve any investigational drugs, vaccines, devices or procedures. All antibiotics used are currently and frequently used antibiotics for ICU-patients. Moreover, the responsible physician can at all times deviate from protocol if clinically indicated. The antibiotic prescription strategies implemented in this trial are interventional but several studies, going back as early as 1985, have used both strategies without any adverse events, especially no increased mortality compared to standard care^6, 9-11, 16, 26^. Consequently, there is no mandated reporting of adverse events.

The study-related procedures applied to human subjects (both HCW and patients in participating ICUs) are considered to be no more than minimal risk procedures.

**8. Ethical considerations**

**Regulation statement**

This study is conducted in agreement with the declaration of Helsinki (Tokyo, October 9th 2004) and with the guidelines of good clinical practice (GCP) issued by the European Union^27, 28^.

**Recruitment and consent**

Study-related procedures involving subjects or subjects’ medical records shall not be initiated prior to initial IRB review and approval. The ICU is enrolled into the study as a unit (“cluster”) upon documented agreement from the ICU director(s) or equivalent. The ICU director(s) will be provided with a copy of the protocol and with enough time to assess the impact of the study on the established procedures of the ICU.

Compliance with Good Clinical Practice (GCP) guidelines for the conduct and monitoring of this clinical trial will occur through observation of the ethical and regulatory requirements of the GCP guidelines of the European Union. By signing this protocol, the investigator agrees to adhere to these requirements. The study protocol will be reviewed and approved by the local IRB or ethics committee. Changes to the protocol will be initiated by the primary investigator and again approved by the local IRB.

The ethical principles and norms of research involving human beings apply to cluster randomized trials, although there is much less published information on the application of these principles and norms to this type of study. A recent publication by the United Kingdom Medical Research Council, “Cluster Randomized Trials: Methodological and Ethical Considerations,” provides guidance in this regard^29^. The approach described below is consistent with the recommendations of this guideline. The physician and nurse director of the ICU will be required to provide written approval for the participation of the ICU in the trial by signing the protocol signature page. This requirement is consistent with the fact that the ICU is the unit of randomization and with the leadership role these individuals play in determining policies and procedures that apply to patients and healthcare workers in the ICU. These leaders will consider the risks and benefits of the strategies and will be required to provide their approval for the enrolment of the ICU and all its admitted patients in the study. They will be held responsible for:

- Implementation and adherence to the antibiotic intervention-strategy,
- Obtaining data regarding patient and ICU characteristics, patient- and ICU-level antibiotic use and HCW hand hygiene protocol compliance,
- (Supervising the) collection of surveillance cultures from ICU patients according to the prescribed schedule

For further information see also the Subcontracting Agreement.

**Informed Consent Waiver**

The justification for a waiver of informed consent from patients in the participating ICUs meets the criteria outlined below.

1. The antibiotics selected represent among the most frequently used agents in ICU patients worldwide, and are in agreement with international guideline recommendations (such as the guidelines from ATS, IDSA and ESICM).

2. The study will involve no more than minimal risk of harm to patients, as physicians can always deviate from treatment protocol if clinically indicated.

3. A waiver will not adversely affect the rights and welfare of patients.

4. The trial cannot practicably be carried out without a waiver.

5. Whenever appropriate, subjects can be provided with additional pertinent information.

Given these considerations, a waiver of informed consent from patients in the participating ICUs is both ethically and statistically justifiable and logistically and economically essential for the proper conduct and analysis of this trial. For the same reasons a waiver of informed consent was provided during a previous cluster-randomized multi-center ICU trial (MOSAR ICU trial), evaluating other approaches to reduce antibiotic resistance in the ICU.

**Benefits and risks assessment**

**Patient and personnel burden**

The burden associated with participation consists of monthly point-prevalence cultures for AMRB for all patients present at that time in ICUs. This will represent only a minority of all patients admitted. One to two hours per month, the RN will do Hand Hygiene Compliance observations, the patient or his or her family can refuse participating in these observations without any consequences.

Physician staff has to be informed and instructed on the use of the two different antibiotic strategies of mixing and cycling.

Principal Investigator and –if not the same person- the Physician-director will supervise the intervention-adherence and general Trial functioning.

**Patient risk**

The risk for patients or HCWs as a result of these interventions is negligible, there is no change in standard-care; antibiotic treatment is still initiated by the treating physician based on local practices and adjusted on microbiological sensitivity data. Moreover, the responsible physician can at all times deviate from protocol if clinically indicated. Benefits for the patients individually and as a group are to be expected primarily in terms of prevention of AMRB acquisition and cross transmission, and secondarily as improvement in patient outcome.

We do not aim to investigate a new procedure by using this intervention. The trial aims to quantify the effect of a known and accepted intervention as part of an Antibiotic Stewardship Program.

**Compensation for injury**

ICUs, HCW, and ICU patients will not be reimbursed for participating in this study. The hospitals will be subcontractors of the SATURN project. ICUs will be compensated for the workload of the RN in exctracting data. The Principal Investigator and/or the UMCU can not be held responsible for any damage to research study subjects through injury or death caused by the study.

The participating centres will provide appropriate insurance which is in accordance with the legal requirements of each country.

**9. Administrative aspects and publication**

**Handling and storage of data and documents**

Anonymized digital databases will be copied and sent to SATURN every 6 months. All electronic data will be stored in secured, SATURN WP2-dedicated non-public electronic databases.

**Confidentiality**

Information linking the patient’s medical data to study materials, will be maintained in a secure location at the site. This information will not be transmitted to the SATURN management or the Principal Investigator and Coordinating Investigator. ICU and individual subject data and records will be held in strictest confidence by the investigator and healthcare staff and by the SATURN project and SATURN-ICU Trial management representatives as permitted by law.

Information on patients and their microbial data will be used and processed solely for the purpose of obtaining outcome measures.

If results from this study are published, the ICUs and individual subject’s identity will remain unknown towards the public, the UMCU and other SATURN-partners.

**Amendments**

Amendments are changes made to the research-protocol after a favourable opinion by the accredited IRB has been given. All amendments will be notified to the IRB(s) that gave a favourable opinion. A ‘substantial amendment’ is defined as an amendment to the terms of the IRB application, or to the protocol or any other supporting documentation, that is likely to affect to a significant degree:

- the safety or physical or mental integrity of the subjects of the trial

- the scientific value of the trial

- the conduct or management of the trial; or

- the quality or safety of any intervention used in the trial

Non-substantial amendments will not be notified to the accredited IRB(s), but will be recorded and filed by the Principal Investigator and Coordinating Investigator.

**Annual progress report**

The Coordinating investigator of the ICU will submit a summary of the progress of the trial to the accredited IRB(s) once a year. Information will be provided on the date of inclusion of the first cluster, numbers of subjects included and numbers of subjects that have completed the trial, other problems, and amendments.

**Public disclosure and publication policy**

Manuscripts and abstracts prepared from the data collected during this trial will be prepared through the study investigators (Principal Investigator and Coordinating Investigator) and the SATURN management. Site investigators will not publish or present interim or definite results (including but not restricted to oral presentations) without written consent of the Principal Investigator. They will of course be allowed to participate in publications regarding this trial. Investigators will provide SATURN with publication or presentation materials in advance of publication/ presentation to allow for review and comment as a means of ensuring confidentiality, accuracy, and objectivity. Timelines for review by SATURN and the Principal Investigator will be laid out in the site list of responsibilities.

According to the rules of the International Committee of Medical Journal Editors, this trial will be registered in a public trial registry^30^.

**12. References**
